# Complete assembly of the organellar genome of *Rubroshorea johorensis* utilizing advanced long-read sequencing technologies

**DOI:** 10.3389/fgene.2025.1574266

**Published:** 2025-05-14

**Authors:** Aditya Nugroho, Evayusvita Rustam, Nurin Widyani, Fitri Indriani, Dede J. Sudrajat, Mohammad Agus Salim, Muhammad Majiidu, Fifi Gus Dwiyanti, Rahadian Pratama, Iskandar Z. Siregar

**Affiliations:** ^1^ Research Center for Applied Botany, National Research and Innovation Agency, Cibinong, West Java, Indonesia; ^2^ Molecular Science Research Group, Advanced Research Laboratory, IPB University, Bogor, West Java, Indonesia; ^3^ Department of Silviculture, Faculty of Forest and Environment, IPB University, Bogor, West Java, Indonesia; ^4^ Department of Biochemistry, Faculty of Mathematics and Natural Science, IPB University, Bogor, West Java, Indonesia

**Keywords:** circular genome, conservation, dipterocarpaceae, MinION, mitogenome, plastome

## 1 Introduction

*Rubroshorea johorensis* (Foxw.) P.S. Ashton and J. Heck, which is a synonym of *Shorea johorensis* Foxw, commonly referred to as red meranti, is a species belonging to the Dipterocarpaceae family (Ashton and Heckenhauer, 2022). This species is found in the tropical rainforests of the Indo-Malayan region (Aisha et al., 2014; Fathiya et al., 2018). *R. johorensis* is naturally distributed across Peninsular Malaysia, Sumatra, and Borneo, particularly in South and East Kalimantan, Brunei, and Central and Western Sarawak (Widiyatno et al., 2014). Typically, *R. johorensis* grows on hillsides at altitudes below 600 m above sea level, on undulating terrain, fertile clayey loam soils, and well-drained alluvial soils (Wahyudi et al., 2014). *Shorea* species, including *R. johorensis*, are essential timber trees known for their high economic value and significant presence in the international tropical timber market (Fathiya et al., 2018; Harahap et al., 2018). These species from the Dipterocarpaceae family are key products of tropical rainforests and serve as symbols of these ecosystems (Widiyatno et al., 2013; Yu et al., 2021). The wood of *R. johorensis* is widely used as a raw material for buildings, furniture, and various other applications (Purwaningsih, 2018; Suciyati et al., 2021).

The IUCN (International Union for Conservation of Nature) Red List of Threatened 2024 categorizes *R. johorensis* as vulnerable (VU) (IUCN, 2024). The population of species within the Dipterocarpaceae family, including the *Shorea* species, *R. johorensis*, has been declining due to logging, the conversion of forest land into plantations, and the establishment of industrial plantations (Widiyatno et al., 2013; Gaveau et al., 2016; Harahap et al., 2018). These activities are contributing to a continuous decrease in the global population of *R. johorensis.* Projections indicate that over three generations (1,860–2,100), the population of this species is expected to decline by 30%–50% (IUCN, 2024). This situation highlights the urgent need for conservation efforts to protect and sustain the existing populations of *R. johorensis*.

Data on the genomic resources of *R. johorensis* are still limited, which poses a significant challenge to advancing research in genetic conservation and forest landscape restoration. Unlocking genomic data for *R. johorensis* is vital for enhancing both genetic conservation strategies and tree breeding programs. Among these genomic resources, organellar genomes are particularly valuable, providing crucial insights into genetic variation among closely related species. In plants, organellar genomes, including mitogenome and plastome, are semi-autonomous structures encased in double membranes, housing independent genetic material. These organelles possess molecular machinery that regulates gene expression (Chevigny et al., 2020) and play a vital role in various physiological processes within plants (Mahapatra et al., 2021).

The plastome is a valuable tool for genetic studies due to its slow evolutionary rate, maternal inheritance in most angiosperms, and its conserved structure and gene sequences (Zulfahmi et al., 2015; Fathiya et al., 2018; Song et al., 2019; Lim et al., 2020). In angiosperms, the plastome typically ranges from 107 kb to 218 kb (Daniell et al., 2016; Li J. et al., 2024) and contains 120 to 130 genes that are crucial for transcription, translation, and photosynthesis (Daniell et al., 2016; Lim et al., 2020). Due to its conserved nature, the plastome is a reliable resource for molecular identification, genetic diversity assessments, and phylogenetic studies (Chew et al., 2023; Kim et al., 2024). In contrast, plant mitogenome exhibit greater variability in size and structural complexity (Burger et al., 2003; Sloan et al., 2012). Their sizes can range from 200 kb to 2,900 kb, with larger genomes typically found in seed plants, which have an abundance of introns, intergenic regions, and repetitive sequences (Mower et al., 2012; Morley et al., 2019). The high rates of sequence repetition, recombination, and rearrangement contribute to the structural diversity of plant mitogenome (Johnston, 2019). Despite these challenges, mitogenome remain invaluable for understanding genome architecture and evolutionary dynamics (Drouin et al., 2008). Therefore, this study aims to sequence, assemble, and characterize the plastome and mitogenome of *Rubroshorea johorensis*. This research enhances understanding of its genetic diversity and contributes to conservation and breeding efforts. It represents a vital step toward preserving this species and enriching the knowledge of plant genetics.

## 2 Methods

### 2.1 Plant material, DNA extraction and sequencing

Fresh leaves of *R*. *johorensis* were collected from the Bogor Botanical Garden in West Java, Indonesia (6° 35′51.46″S, 106° 47′58.44″E), specifically from plot XXV, with collection number 237. The herbarium voucher for this sample has been deposited in the Herbarium Bogoriense (BO) with passport data BO 1997911. A total of 100 mg of fresh leaves was used for genomic DNA isolation, following the CTAB protocol (Doyle and Doyle, 1990). The initial quality of the extracted genomic DNA was assessed by visualizing it on a 1% agarose gel electrophoresis in TAE buffer, run for 30 min at 100 V. Subsequently, the purity and quantity of the extracted genomic DNA were determined using a NanoPhotometer^®^ NP80 (IMPLEN) and a Qubit 1.0 Fluorometer (Thermo Fisher Scientific), utilizing the Qubit dsDNA BR assay kit. The long-read sequencing library was prepared according to the protocol provided by Oxford Nanopore Technologies (ONT) for Ligation Sequencing DNA V14 (SQK-LSK114), version ACDE_9163_v114_revQ_29 Jun 2022. Sequencing was conducted using a MinION Mk1C device equipped with an R10.4.1 flow cell (FLO-MIN114). All laboratory work was carried out at Forest Genetics and Molecular Forestry, Department of Silviculture, Faculty of Forestry and Environment, IPB University in Bogor, West Java.

### 2.2 Organelle genome assembly and annotation

Raw reads from MinION sequencing (POD5) were base-called into raw Fastq files using Dorado Basecaller v0.8.0. The resulting Fastq files were subsequently processed using Porechop v0.2.4 (Wick et al., 2017) and Chopper v0.5.0 (De Coster, 2018) to trim adaptors, remove low-quality bases (Phred score <9), and eliminate potential sequence contamination. The parameters used for Chopper included -l 500, -q 9, --head crop 10, and–tail crop 10. The cleaned Fastq data were then analyzed statistically using NanoPlot v1.31.0 (De Coster et al., 2018) to calculate and visualize the read distribution.

The *de novo* assembly of the *R. johorensis* organelle genome was performed using Flye v2.9.4 (Kolmogorov et al., 2019) to construct both the plastome and mitogenome. To enhance accuracy, the assembled contigs were polished using the Pilon v1.20.1 (Walker et al., 2014). These contigs were then mapped to the reference plastome of *Shorea macrophylla*, a synonym of *Rubroshorea macrophylla* (GenBank accession: ON321899), and to the mitogenome of *Arabidopsis thaliana* (GenBank accession: GCF_000001735.4).

The annotation was carried out using CPGAVAS2 (http://47.96.249.172:16019/analyzer/annotate) (Shi et al., 2019), with the plastome of *S. macrophylla* (accession number: ON321899) serving as the reference. The results of the annotation were manually verified using Unipro UGENE v45.1 (Okonechnikov et al., 2012) and NCBI Genomic Workbench v3.8.2 (Kuznetsov and Bollin, 2021). For the mitogenome, annotation was conducted using PMGA (http://47.96.249.172:16084/annotate.html) (Li L. F. et al., 2024). These results were also manually verified using Unipro UGENE v45.1 and NCBI Genomic Workbench v3.8.2. The circular genome was visualized using Organellar GenomeDRAW (OGDRAW), accessible through the MPI-MP Chlorobox platform (Greiner et al., 2019).

## 3 Result

Long-read sequencing of *R. johorensis* yielded a total of 1,657,747 reads, amounting to 5,759, 232, 230 base pairs (bp) of raw data. The mean read length was 3,474.1 bp, and the N50 value for read length was 5,104 bp. The mean read quality of the raw data was recorded as 12.7. After filtering, the number of reads that passed the quality assessment reached 1,463,146, totaling 5,198,148,860 bp. The mean read length increased to 3,552.7 bp, with the N50 value remaining stable at 5,105 bp. Additionally, the mean read quality improved to 15.1 after filtering. A total of 29,886 reads (30.94%) were successfully mapped to the plastome, resulting in a mean depth of coverage of 104.4×. For the mitogenome, mapping of sequencing reads to the *A. thaliana* reference genome resulted in a mean coverage depth of 44×, with 23.4% of the genome covered by at least one read. In contrast, the assembly of the *R. johorensis* mitogenome yielded an average coverage depth of 31×, indicating reliable sequencing depth across the majority of the mitochondrial genome.

**FIGURE 1:**
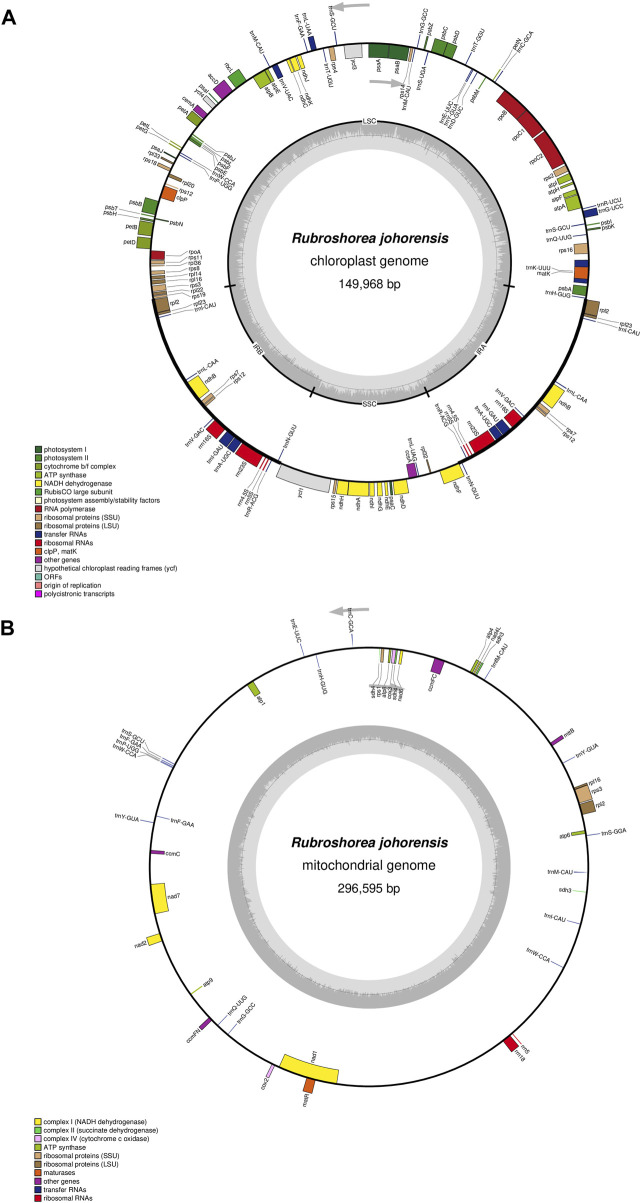
The organelle genome map of *Rubroshorea johorensis*. **(A)** The circular map of plastome genome in *R. johorensis*. **(B)** The circular map of the mitogenome in *R. johorensis*.

The assembled plastome of *R. johorensis* has a total length of 149,968 bp, while its mitogenome spans 296,595 bp, with a GC content of 44.63% (Figure 1). The plastome structure consists of a Large Single Copy (LSC) region of 82,920 bp with a GC content of 35.39%, a Small Single Copy (SSC) region of 19,837 bp with a GC content of 31.55%, and two Inverted Repeat (IR) regions, each measuring 23,584 bp, with a GC content of 43.36%. Among these regions, the IR regions exhibited the highest GC content. Overall, the average GC content of the plastome was 37.39%.

Annotation of the *R. johorensis* plastome identified 126 genes, comprising 76 protein-coding genes, 29 tRNA genes, and four rRNA genes. Among them, 15 genes contain a single intron, while three genes (*rps*12, *ycf*3, and *clp*P) contain two introns. These genes were categorized into four functional groups: self-replicating genes, photosynthetic genes, genes with other functions, and genes of unknown function (Supplementary Table S1).

In the mitogenome of *R. johorensis*, a total of 44 genes were identified, which include two rRNA genes, 17 tRNA genes, and 25 protein-coding genes. Additionally, six of these genes contain introns, suggesting that RNA processing events may play a crucial role in gene expression and regulation, with further details provided in Supplementary Table S2. Similar to the mitogenome of other plants, *R. johorensis* likely exhibits high rates of recombination and structural rearrangements, features that are characteristic of plant mitogenome and contribute to genome plasticity. The number of protein-coding genes (22) in *R. johorensis* is comparable to that found in other land plants, supporting the idea that core mitochondrial functions are conserved across species. However, variations in gene content and the presence of introns may indicate species-specific adaptations that could be linked to environmental factors or evolutionary history. The results of the mitogenome sequencing revealed that the assembled genome is still limited. This finding is significant as this study represents the first comprehensive characterization of the mitogenome for a species within the Dipterocarpaceae family. It underscores the need for further research in this area. Conducting additional comparative genomic studies could provide deeper insights into the functional implications of these genomic features in *R. johorensis*.

**FIGURE 2:**
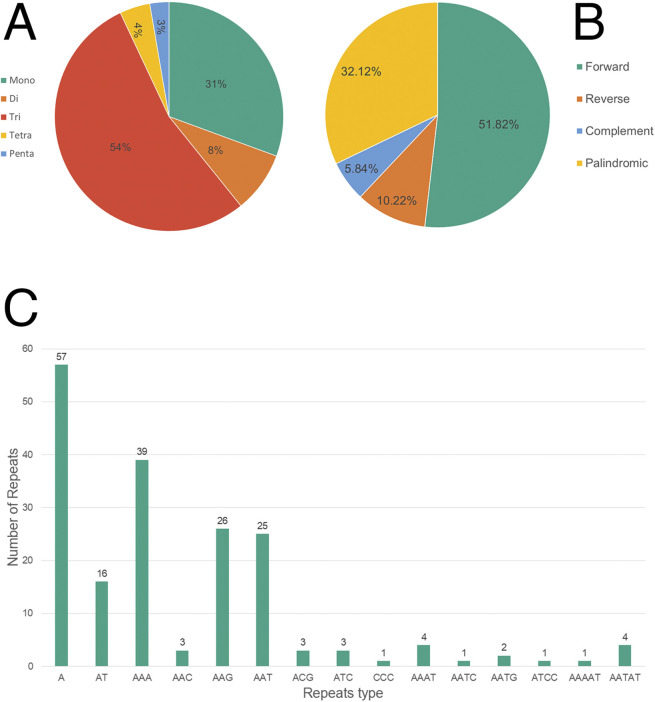
Frequency of simple sequence repeat (SSR) and repetitive regions in the *Rubroshorea johorensis* plastome. **(A)** Number of different SSRs types. **(B)** Distribution of repetitive regions. **(C)** Number of different SSRs motifs.

Microsatellites (SSRs), or simple sequence repeats (1–6 bp), are widely distributed throughout genomes and hold significant importance in genomic analysis. This study identified various types of SSRs in the genome, with trinucleotide repeats being the most prevalent at 54%, followed by mononucleotide repeats at 31%. Dinucleotide, tetranucleotide, and pentanucleotide repeats were less common, comprising 8%, 4%, and 3%, respectively. Adenine-rich sequences were particularly abundant, with A (57), AAA (39), AAG (26), and AAT (25) emerging as the most frequent types. The analysis of longer repeats showed a dominance of forward repeats (51.82%), followed by palindromic repeats (32.12%). Reverse and complementary repeats were less prominent, representing for 10.22% and 5.84%, respectively (Figure 2).

## Data Availability

The datasets presented in this study can be found in online repositories. The names of the repository/repositories and accession number(s) can, be found below: https://www.ebi.ac.uk/ena/browser/view/PRJEB73584, https://www.ebi.ac.uk/ena/browser/view/SAMEA115502444, https://www.ebi.ac.uk/ena/browser/view/ERR13245837, https://www.ncbi.nlm.nih.gov/genbank/, PV078022, https://www.ncbi.nlm.nih.gov/genbank/, PV540234.
