# Supplementary material for: Complete assembly of the organellar genome of Rubroshorea johorensis utilizing advanced long-read sequencing technologies
Source: Front Genet. 2025 May 14;16:1574266. doi: 10.3389/fgene.2025.1574266 (PMC12116502; doi:10.3389/fgene.2025.1574266)
Supplement: Supplementary file 1 [file Supplementaryfile1.docx]

Supplementary Material

# Supplementary Tables

**Table 1 Gene content in plastome of *Rubroshorea johorensis***

| **Functional category** | **Group of gene** | **Gene** |
| --- | --- | --- |
| Self-replication | rRNA | rrn16S (2x), rrn23S (2x), rrn5S (2x), rrn4.5S (2x) |
|  | tRNA | trnA-UGC* (2x), trnC-GCA, trnD-GUC, trnE-UUC, trnF-GAA, trnG-GCC, trnG-UCC*, trnH-GUG, trnI-CAU, trnI-GAU* (2x), trnK-UUU*, trnL-CAA (2x), trnL-UAA*, trnL-UAG, trnM-CAU (2x), trnN-GUU (2x), trnP-UGG, trnQ-UUG, trnR-ACG (2x), trnR-UCU, trnS-GCU (2x), trnS-UGA, trnT-GGU, trnT-UGU, trnV-GAC (2x), trnV-UAC* ,trnW-CCA, trnY-GUA |
|  | Subunits of ATP synthase | atpA, atpB, atpE, atpF, atpH, atpI |
|  | Subunits of photosystem II | psbA, psbB, psbC, psbD, psbE, psbF, psbI, psbJ, psbK, psbL, psbM, psbN, psbT, psbZ, ycf3** |
|  | Subunits of NADH-dehydrogenase | ndhA*, ndhB* (2x), ndhC, ndhD, ndhE, ndhF*, ndhG, ndhH, ndhI, ndhJ, ndhK |
| Photosyntesis | Subunits of cytochrome b/f complex | petA, petB*, petD*, petG, petL, petN |
|  | Subunits of photosystem I | psaA, psaB, psaC, psaI, psaJ |
|  | Subunit of rubisco | rbcL |
|  | Large subunit of ribosome | rpl14, rpl16, rpl2* (2x), rpl20, rpl22, rpl23 (2x), rpl32, rpl33, rpl36 |
|  | DNA-dependent RNA polymerase | rpoA, rpoB, rpoC1*, rpoC2 |
|  | Small subunit of ribosome | rps11, rps12 (2x), rps14, rps15, rps16*, rps18, rps19, rps2, rps3, rps4, rps7 (2x), rps8 |
|  | Subunit of Acetyl-CoA-carboxylase | accD |
|  | c-type cytochrome synthesis gene | ccsA |
| Other function | Envelope membrane protein | cemA |
|  | Protease | clpP** |
|  | Maturase | matK |
| Conserved open reading frames | Conserved open reading frames | ycf1*, ycf4 |

2x, gene duplication; *, single intron; **, double intron.

**Table 2 Gene content in mitogenome of *Rubroshorea johorensis***

| **Functional category Group of gene** | | **Gene** |
| --- | --- | --- |
| rRNA genes | rRNA | rrn5, rrn18 |
| tRNA genes | tRNA | trnC-GCA, trnE-UUC, trnF-GAA (2x), trnfM-CAU, trnG-GCC, trnH-GUG, trnM-CAU, trnP-UGG, trnQ-UUG, trnS-GCU, trnS-GGA, trnW-CCA (2x), trnY-GUA (2x) |
| Variable genes | Succinate dehydrogenase | sdh3 (2x), sdh4 (2x) |
|  | Large subunit of ribosome | rpl2*, rpl16 |
|  | Small subunit of ribosome | rps1, rps3* |
| Core genes | ATP synthase | atp1, atp4, atp6, atp8, atp9 |
|  | Cytochrome c biogenesis | ccmFC*, ccmFN, ccmC |
|  | Cytochrome c oxidase | cox2, cox3 |
|  | Maturases | matR |
|  | Transport membrane protein | mttB |
|  | NADH dehydrogenase | nad1**, nad2**, nad4L, nad6, nad7** |

2x, gene duplication; *, single intron; **, double introns
